# Increasing blood pressure and its associated factors in Canadian children and adolescents from the Canadian Health Measures Survey

**DOI:** 10.1186/1471-2458-12-388

**Published:** 2012-07-12

**Authors:** Yipu Shi, Margaret de Groh, Howard Morrison

**Affiliations:** 1 Science Integration Division, Center for Chronic Disease Prevention and Control, Public Health Agency of Canada, 785 Carling Avenue, Ottawa, ON, Canada, K1A 0K9

## Abstract

**Background:**

Canada is facing a childhood obesity epidemic. Elevated blood pressure (BP) is a major complication of obesity. Reports on the impact of excess adiposity on BP in children and adolescents have varied significantly across studies. We evaluated the independent effects of obesity, physical activity, family history of hypertension, and socioeconomic status on BP in a nationally representative sample of children and adolescents.

**Methods:**

We analysed cross-sectional data for 1850 children aged 6 to 17 years who participated in the Canadian Health Measures Survey, Cycle 1, 2007–2009. Systolic BP (SBP) and diastolic BP (DBP) were age-, sex-, and height-adjusted to z-scores (SBPZ and DBPZ). Body mass index (BMI) z-scores were calculated based on World Health Organization growth standards. Multivariate linear regression was used to evaluate the independent effects of relevant variables on SBPZ and DBPZ.

**Results:**

For most age/sex groups, obesity was positively associated with SBP. Being obese was associated with higher DBP in adolescent boys only. The BP effect of obesity showed earlier in young girls than boys. Obese adolescents were estimated to have an average 7.6 mmHg higher SBP than normal weight adolescents. BMI had the strongest effect on BP among obese children and adolescents. Moderately active adolescent boys had higher SBP (3.9 mmHg) and DBP (4.9 mmHg) than physically active boys. Family history of hypertension showed effects on SBP and DBP in younger girls and adolescent boys. Both family income and parent education demonstrated independent associations with BP in young children.

**Conclusions:**

Our findings demonstrate the early impact of excess adiposity, insufficient physical activity, family history of hypertension, and socioeconomic inequalities on BP. Early interventions to reduce childhood obesity can, among other things, reduce exposure to prolonged BP elevation and the future risk of cardiovascular disease.

## Background

Between 1978/9 and 2004, the prevalence of obesity among Canadian children aged 2 to 17 years doubled from 6.3 % to 12.7 %, while rates of overweight increased from 23.3 % to 34.7 % [1]. One of the major complications of obesity, elevated blood pressure (BP), is found increasingly in Canadian children [2,3]. BP tracks with age and elevated BP at a young age predicts essential hypertension in adulthood [4]. From a public health perspective, understanding factors associated with increasing BP in Canadian children can help inform interventions to reduce risks and optimize BP during childhood.

Many studies have shown a positive association between obesity and hypertension in children of various populations [5,6]. The definition of hypertension in children is based on percentile cutoffs from normative distribution of BP in children presumed healthy [7]. There have been concerns that these statistically derived thresholds may underestimate children at risk [8]. BP is a continuous variable that is positively correlated with cardiovascular risk across the entire BP range [9,10]. Race/ethnicity is another factor associated with differences in the prevalence of hypertension and the degree of arterial stiffness associated with high BP in children [11,12]. Thus, it is prudent to have population-specific evaluation of childhood BP and its associated factors for the full range of BP levels.

Health behaviours established in childhood tend to continue into adulthood. Most studies support the beneficial effect of physical activity (PA) in lowering BP in children [13,14], although unfavourable effects of moderate to vigorous intensity PA on BP have also been reported [15,16]. Family history of hypertension (FHH) is a particularly important factor associated with high BP risk and its influence on BP occurs well before adolescence [17]. Although FHH cannot be modified, we do not yet know if children genetically predisposed to hypertension respond differently to excess body weight or other factors with regard to their BP. Furthermore, while socioeconomic inequalities are linked to childhood obesity in western developed countries [18], there are no reports on their impact on BP among children.

Several studies have reported on the prevalence of hypertension and pre-hypertension in Canadian children, but results are inconsistent [2,3]. Because of BP variability and suboptimal BP measurements, guidelines for minimum standards for surveys measuring BP were developed in Canada [19]. The Canadian Health Measures Survey (CHMS) is the first national survey to measure BP using these guidelines [20]. The goal of our study was to evaluate the independent effects of obesity, PA, FHH and socioeconomic status (SES) on systolic BP (SBP) and diastolic BP (DBP) according to age and gender in a nationally representative sample of children and adolescents.

## Methods

We used cross-sectional data from the CHMS, cycle 1, 2007–2009, a complex sampling survey designed to collect data on self-reported and direct measures of health and wellness from a representative sample of approximately 5600 Canadians aged 6 to 79 years. The CHMS covers approximately 96.3 % of the Canadian population living in private dwellings in all ten provinces and three territories, but excludes those living on reserves and certain remote areas, institutional residents and full-time members of the Canadian Forces. Health Canada’s Research Ethics Board reviewed and approved all processes and protocols for cycle 1 of the CHMS. Informed consent was obtained from all participants before starting any study procedures. Overall, the combined response rate was 51.7 % for cycle 1 of the CHMS [20].

The CHMS cycle 1 included data for 1878 participants aged 6 to 17 years. For our purposes, we excluded 7 respondents missing BP measures, 2 missing BMI measures, and one being treated with hypertension medication. All study respondents were cross-tabulated for conditions that are the primary causes of BP elevation; one reported having diabetes mellitus type 1 and one other diabetes mellitus type 2; 11 reported congenital heart disease; and 4 reported kidney dysfunction or disease. We also excluded them from our analyses. Two participants who reported having hypertension but were not being treated with medications were retained. After an initial exploration of the data, we also excluded one 9-year-old boy whose BP was 173/127 mmHg, for which an underlying condition was suspected. Thus, the final analyses included data for 1850 participants.

The survey consisted of a personal household interview followed by a physical examination at a mobile examination center within 2 days to 6 weeks of the interview. The household interview included a questionnaire about general demographic information and an in-depth health questionnaire. Parents/guardians answered all the applicable household questions for participants aged 6 to 13 years and, with assistance from children responded to questions about, for example, participation in physical activity during schools hours. Participants aged between 14 and 17 years answered all questions on their own.

Standing height and weight were measured and used to calculate BMI (weight [kilograms] divided by height [meters] squared). Age- and sex-adjusted BMI z-scores (BMIZ) were calculated based on World Health Organization (WHO) child growth charts [21]. According to WHO, obesity is defined as a BMIZ that is more than two standard deviations (SDs) above the mean (≈ 97.7^th^ percentile) and overweight as a BMIZ between one and two SDs above the mean (≈ 84^th^ to 97.7^th^ percentile).

Participants’ resting BP was measured according to the new protocol for standard BP measurements in surveys [19]. Participants were invited to wait in a quiet and comfortable environment for at least 5 minutes before the measurement. BP was measured using an automated BP cuff (BpTRU) on the right arm with a cuff size suitable for the arm circumference. Six BP readings were taken with a clinically validated oscillometric device (BpTRU^TM^ BP-300, BpTRU^TM^ Medical Devices Ltd., Coquitlam, British Columbia) at 1-minute intervals, with BP determined as the average of the last 5 measurements. Heart rate was measured in beats per minute (bpm) with each BP measurement and averaged using the last 5 measurements. BP was measured manually only when inconsistency was related to high variability or too many errors, which occurred in fewer than 1 % of cases. More detailed information on BP measurement in this survey can be found elsewhere [22].

We calculated age-, sex- and height-adjusted SBP z-scores (SBPZ) and DBP z-scores (DBPZ) using the US reference data [7]. We defined systolic or diastolic hypertension as SBPZ or DBPZ at or above the 95^th^ percentile. Pre-hypertension was defined as SBPZ or DBPZ between the 90^th^ and 95^th^ percentile or as for adults (≥120/80 mmHg).

The PA model for adults was adopted for adolescents aged 12 to 17 years [23]. Survey participants or their parents were asked about the type, frequency and duration of PA in the past 3 months during leisure time and at school. Energy expenditure (EE) was calculated using the frequency and duration per session of the PA as well as metabolic equivalents (MET) value of the activity. Calculated total daily EE values were used to develop a PA index: active, moderately active, and inactive. The PA of children aged 6 to 11 years was determined from the answers given to the question “Over a typical or usual week, on how many days was he/she physically active for a total of at least 60 minutes per day?” If the answer was “4 days or more”, the child was regarded as physically active; otherwise, the child was considered inactive.

We determined FHH from answers to the questions “Has anyone in your immediate family ever had high BP, excluding during pregnancy?” and “What is the youngest age at which a member of your immediate family was first diagnosed with high BP?” Participants who answered “Yes” to the former and reported that age at diagnosis was less than 60 years were considered to have an FHH.

We determined the highest education achieved by adult members of the household and dichotomized this based on whether at least one adult had completed post-secondary education. Household income was categorized into two groups (low, middle or high) based on the total family income from all sources and the number of people in the household; a third category was created for those with data missing on this variable. We defined ethnicity as whites and non-whites including Aboriginals living off reserves. Children aged 12 years and older were asked about cigarette smoking.

Because BP may increase substantially with the onset of puberty and between sexes [24], we stratified analyses for children (6 to 11 years) and adolescents (12 to 17 years), and for boys and girls. Associations of SBPZ or DBPZ with overweight and obesity, PA, FHH, education and income were estimated simultaneously using multivariate linear regression models, controlling for covariates - age and heart rate. Variables of ethnicity and cigarette smoking (adolescents only) were initially included in the models, but dropped later because they led no changes to the results. Another consideration in model construction was the 11 degrees of freedom requirement in statistical analysis, which restricts the number of parameters that can be entered into a model to 10. To evaluate the possible interactions between BMIZ and FHH on BPZ, we added an interaction term for BMIZ and FHH to the full regression model. To evaluate the potentially varying effects of BMIZ on BPZ with respect to normal weight, overweight and obesity, we stratified analyses according to BMI category and performed multivariate linear regressions within each stratum. We were not able to carry out subgroup analysis for each stratum because of limited sample size.

The bootstrap method, which takes into account the complex survey design, was used to estimate standard errors, coefficients of variation and confidence intervals for means, proportions and coefficients of regression parameters. Because of restricted primary sampling units, Statistics Canada requires that 11 *df* be applied to all the variance estimation with bootstrap method. Statistical significance in all analyses was defined as a p value <0.05. The analyses were performed using SAS Enterprise Guide 4 software (SAS Institute Inc.) and Bootvar 3.2 SAS version (Statistics Canada; http://www.statcan.gc.ca/rdc-cdr/bootvar_sas-eng.htm).

## Results

### Population characteristics

Overall, both mean SBP and DBP were markedly higher in adolescents (98.7 mmHg and 62.5 mmHg, respectively) than in children (93.2 and 60.5 mmHg) (Table 1). Mean SBPZ was −0.7 (95 % CI, -0.75,-0.65) in children and −1.2 (95 % CI, -1.38,-1.08) in adolescents, suggesting lower SBP for Canadian children compared to US counterparts. Mean DBPZ in children, 0.07 (95 % CI, 0.01,0.13) was higher than US children, mean DBPZ in adolescents, -0.22 (95 % CI, -0.34,-0.10) was lower than US adolescents. The prevalence of hypertension was less than 1 %, and the prevalence of pre-hypertension was about 2.2 %. More than 30 % of young people were either overweight or obese (13 % of children and 15 % of adolescents were obese, while another 20 % of children and 16 % of adolescents were overweight). A majority of children (83.6 %) and just over half of adolescents (55.2 %) reported being physically active, while 13.9 % of children and 20.5 % of adolescents reported having FHH. About 7 % of participants were from low-income families, and at least one parent of over 70 % of study participants had post-secondary education. Around three-quarters (75.5 %) of study subjects were white, and 6.9 % of adolescents reported smoking cigarettes daily.

**Table 1:** Characteristics of Canadian children and adolescents in the Canadian Health measures Survey, 2007–2009

	Age, years					
	6 to 11 (n = 1058)			12 to 17 (n = 792)		
	n	Mean or %	95 % CI	n	Mean or %	95 % CI
Boys, n (%)	531	50.5	49.7, 51.4	416	54.2	51.9, 56.5
Mean systolic blood pressure (mmHg)	1058	93.2	92.4, 93.9	792	98.7	97.0, 100.3
Mean diastolic blood pressure (mmHg)	1058	60.5	59.7, 61.4	792	62.5	61.2, 63.9
Mean systolic blood pressure z-scores	1058	−0.70	−0.75,-0.65	792	−1.20	−1.38, -1.08
Mean diastolic blood pressure z-scores	1058	0.07	0.01, 0.13	792	−0.22^e^	−0.34, -0.10
Mean heart rate (bpm)	1058	75	74, 77	792	80	78, 81
Mean body mass index (kg/m^2^)	1058	17.8	17.6, 18.0	792	22.0	21.1, 23.0
Mean body mass index z-scores (BMIZ)	1058	0.51	0.41, 0.61	792	0.47^e^	0.22, 0.73
Body mass index category, n (%)						
Normal weight (BMIZ < 84 percentile)	733	67	63.2, 70.7	558	69.2	61.1, 77.3
Overweight (84 ≤ BMIZ < 97.7 percentile)	195	20.2	14.9, 25.4	143	15.8	11.6, 19.9
Obese (BMIZ ≥ 97.7 percentile)	131	12.9	9.9, 15.8	91	15.1^e^	8.6, 21.6
Physically active, n (%)	888	83.6	80.5, 86.6	424	55.2	49.3, 61.2
Family history of hypertension, n (%)	161	13.9	11.2, 16.5	173	20.5	16.8, 24.2
Low family income, n (%)	74	6.9^e^	3.6, 10.2	43	7.0^e^	3.2, 10.8
Post-secondary educated parents, n (%)	856	79.9	74.2, 85.6	605	73.1	65.5, 80.8
Ethnicity - whites, n (%)	814	75.4	62.0, 88.7	635	75.6	65.6, 85.6
Daily smoking, n (%)	–	–	–	53	6.9^e^	2.1, 11.7

Mean and proportion (%) were estimated using bootstrap method; CI, confidence interval.

e, CV coefficient of variation of 16.6 % − 33.3 %, interpret with caution.

### Factors associated with increasing SBP and DBP

Table 2 shows the associations of interested variables with SBPZ and DBPZ in children. BMIZ was significantly associated with SBPZ in young girls (*β* = .09; *p* = .02). Every 1-unit increase in BMIZ in young girls is associated with higher SBPZ by .09SD, equivalent to ~ .9 mmHg higher SBP (not shown in the table). When categorized BMI were used in the model, higher SBPZ were found for overweight (*β* = .20; *p* = .04) and obese (*β* = .34; *p* = .004) young girls compared with normal weight young girls. Obesity in young girls was associated with ~3.5 mmHg higher SBP. Neither BMIZ nor being obese was associated with DBPZ in either sex. FHH was positively associated with increases in both SBPZ (*β* = .31) and DBPZ (*β* = .26) for young girls, equivalent to ~3.6 mmHg higher SBP and ~3.1 mmHg higher DBP, but not in young boys. As expected, young girls whose parents had post-secondary education had, on average lower SBPZ. However, low family income was significantly associated with lower SBPZ in young boys and lower DBPZ in young girls. Age was inversely associated with SBP and DBP in both young boys and girls. Heart rate was significantly associated with both SBP and DBP in children.

**Table 2:** Associations of systolic blood pressure (SBP) and diastolic blood pressure (DBP) with relevant variables in children (ages 6–11) according to gender, Canadian Health Measures Survey, 2007–2009

	SBP z-scores		DBP z-scores	
	*β*	*p*	*β*	*p*
Boys (n = 530)				
Body mass index category - Normal weight	1		1	
- Overweight	.07	.36	.07	.29
- Obese	.21	.15	-.02	.90
Heart rate	.01	.009	.02	<.001
Family history of hypertension – No	1		1	
- Yes	.14	.22	.06	.40
Physical activity – Active	1		1	
- Inactive	-.12	.37	-.11	.34
Ages	-.05	.02	-.04	.02
Family income – Middle or high	1		1	
- Low	-.19	.02	-.10	.44
Parental post-secondary education – No	1		1	
- Yes	-.11	.21	-.10	.36
*R*^2^	.13		.15	
Girls (n = 528)				
Body mass index category – Normal weight	1		1	
- Overweight	.20	.04	.03	.74
- Obese	.34	.004	-.09	.44
Heart rate	.02	<.001	.02	<.001
Family history of hypertension - No	1		1	
- Yes	.31	.008	.26	.03
Physical activity - Active	1		1	
- Inactive	-.003	.96	-.17	.01
Ages	-.13	<.001	-.08	.002
Family income - Middle or high	1		1	
- Low	-.23	.10	-.42	<.001
Parental post-secondary education - No	1		1	
- Yes	-.17	.02	-.14	.17
*R*^2^	.18		14	

Dependent variable: SBP z- scores or DBP z-scores. All variables in the left column of the table were independent variables, and their associations with SBP or DBP were simultaneously adjusted using multivariate linear regression models. *β,* coefficient of regression parameters; *p:* <0.05.

Table 3 shows the associations of interested variables with increasing SBPZ and DBPZ in adolescents. BMIZ was significantly associated with SBPZ in adolescent boys (*β* = .22; *p* = .001). Every 1-unit incremental increase in BMIZ in adolescent boys increased SBPZ by .22SD, equivalent to ~2.4 mmHg increase in SBP (not shown). Compared to normal weight adolescents, significantly higher SBPZ was observed among both obese adolescent boys and girls (*β* = .69 and 0.74, respectively, equivalent to ~7.6 mmHg and ~7.7 mmHg). Obese adolescent boys also demonstrated markedly higher DBPZ (*β* = .21; *p* = .04), equivalent to ~2.8 mmHg higher DBP. Compared to active PA, moderate PA was significantly associated with increases in both SBPZ and DBPZ in adolescent boys (SBPZ: *β* = .34, *p* = .02; DBPZ: *β* = .38, *p* < .001), equivalent to ~3.9 mmHg for SBP and ~4.9 mmHg for DBP. Moreover, FHH in adolescent boys was positively associated with both SBPZ and DBPZ (SBPZ: *β* = .44, *p* = .009; DBPZ: *β* = .30, *p* = .02), equivalent to ~4.6 mmHg for SBP and ~3.1 mmHg for DBP. Age was inversely associated with SBP and DBP in adolescent boys, whereas positive effect of age on DBP was observed for adolescent girls. Heart rate was positively associated with DBP in adolescents. Of note, the overall adjusted *R*^2^ was estimated at 0.75 for SBP in adolescent boys, indicating that the variables included in the model explained three-quarters of BP variability in this population.

**Table 3:** Associations of systolic blood pressure (SBP) and diastolic blood pressure (DBP) with relevant variables in adolescents (ages 12–17) according to gender, Canadian Health Measures Survey, 2007–2009

	SBP z-scores		DBP z-scores	
	*β*	*p*	*β*	*p*
Boys (n = 416)				
Body mass index category - Normal weight	1		1	
- Overweight	.38	.01	.21	.19
- Obese	.69	<.001	.21	.04
Heart rate	.006	.31	.009	.03
Family history of hypertension - No	1		1	
- Yes	.44	.009	.30	.02
Physical activity - Active	1		1	
- Moderate active	.34	.02	.38	<.001
- Inactive	.23	.12	.21	.06
Ages	−.08	.001	−.07	<.001
Parental post-secondary education - No	1		1	
- Yes	−.16	.20	−.09	.39
*R*^2^	.75		.24	
Girls (n = 376)				
Body mass index category - Normal weight	1		1	
- Overweight	.09	.52	−.01	.93
- Obese	.74	<.001	.17	.19
Heart rate	.009	.06	.01	.007
Family history of hypertension - No	1		1	
- Yes	.16	.20	.09	.42
Physical activity - Active	1		1	
- Moderate active	.03	.77	−.06	.61
- Inactive	−.10	.35	−.20	.12
Ages	−.004	.85	.05	.01
Parental post-secondary education - No	1		1	
- Yes	−.12	.38	−.07	.51
*R*^2^	.18		.12	

Dependent variable: SBP z- scores or DBP z-scores. All variables in the left column of the table were independent variables, and their associations with SBP or DBP were adjusted simultaneously using multivariate linear regression models. *β,* coefficient of regression parameters; *p:* <0.05.

### Interaction by BMI and FHH on the association of BMI with BP

We examined the interaction term between BMIZ and FHH in the multiple linear regression model for the whole sample. No statistical significance was found for this interaction term (*β* = .005; *p* = .93).

### Differences in association between BP and BMI by BMI category

**Figure 1:**
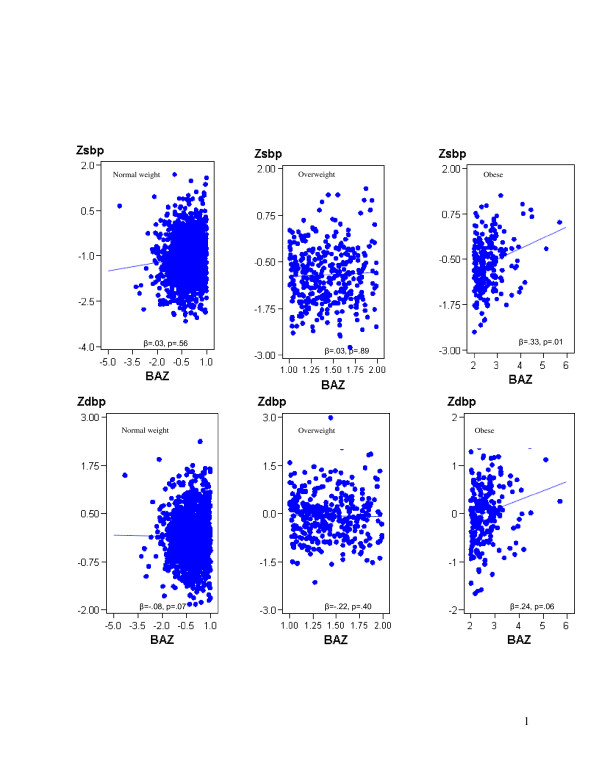
**Short title: Varying effects of BMI on blood pressure by childhood obesity status**. Legend of Figure 1: Varying effects of BMI z-scores (BAZ) on SBP and DBP z-scores (Zsbp and Zdbp), stratified by weight status: normal weight, overweight, and obesity (WHO cut-points), controlling for age, sex, physical activity, familial history of hypertension, parents’ education and family income. β, coefficient of regression parameters (slope of the regression line); *p*, <0.05. Canadian Health Measures Survey, 2007–2009.

We sought to determine whether the effect of BMI on BP varies among normal weight, overweight and obese children and adolescents. We found that BMI was neither associated with SBP (*β* = .03; *p* = .56), nor with DBP (*β* = −.08; *p* = .07) in the normal weight group, nor in the overweight group (SBP: *β* = .03, *p* = .89; DBP: *β* = −.22, *p* = .40). By contrast, there was a strong association of BMI with SBP (*β* = .33; *p* = .02), and a borderline association with DBP (*β* = .24; *p* = .06) among obese group (Figure 1).

## Discussion

The current study examined the association of childhood BP and its determinants in a nationally representative sample of Canadian children and adolescents. We found that obesity was positively associated with SBP, but not with DBP (except in adolescent boys among whom obesity was associated with higher DBP). We observed that obese adolescents had an average of 7.6 mmHg higher SBP than that of normal weight adolescents. BMI had the strongest effect on BP among obese children and adolescents. Less physically active adolescent boys had higher SBP (3.9 mmHg) and DBP (4.9 mmHg) than physically active boys. FHH in young girls and adolescent boys was strongly associated with increases in both SBP and DBP. Both family income and parental education played independent roles on BP in young children.

Based on the first national physical measures survey, we reported that the prevalence of childhood hypertension and pre-hypertension was at 2-3 %, which is significantly lower than other Canadian studies, in which prevalence of hypertension was reported at 7.4 % and 14 % [2,3]. However, it is difficult to compare across studies because of differences in age, study populations (i.e. rural resident) and variability in BP measurement methods. Our results do seem to be in line with those studies with repeated BP measurements [25,26], suggesting the importance of standardizing BP measures. Our observations that BMI or obesity is consistently associated with increasing SBP are consistent with a series of studies performed on various populations, though some studies also observed the effect of BMI on DBP [2,6]. The mechanism underlying the association between obesity and hypertension is poorly understood. Sorof et al. proposed that obesity-induced hypertension may be mediated in part by sympathetic nervous system hyperactivity, which is partly manifested by increased heart rate and BP variability [6]. This possibility is supported by our data that heart rate is positively associated with BP, especially among children, and that a significant difference in heart rate was observed in the obese compared with nonobese groups (data not shown). Most research has focused on SBP because it is a strong predictor of arterial stiffness and many other cardiovascular risks growing into adulthood [11]. Our results support the close association between SBP and obesity, reinforcing the importance of maintaining a healthy weight for optimal SBP during childhood. In addition to substantial increases in SBP, we found markedly higher DBP in obese adolescent boys. This might be explained by evidence of early atherosclerosis at autopsy and ultrasound findings of the carotid artery, in which severely obese children, especial boys, display arterial stiffness and increased diastolic wall stress, indicating adverse changes in vascular health that could be the culprit for the increases in both SBP and DBP [27].

Furthermore, our results show that BMI has the strongest effect on BP among obese children and adolescents. Rosner et al. proposed that there may be a different effect of BMI on BP depending on the differing levels of fatness among various ethnic groups [12]. Our result are consistent with a recent study by Tu et al. who found that there is a marked intensification of the influence of adiposity on BP when children reach the categories of overweight and obese [28]. Moreover, the differing effect of BMI on both SBP and DBP may well be explained by some intervention studies. One such study by Rocchini et al. demonstrated that weight loss among obese adolescents changed the BP distribution from right skewed to no difference from the general population; however, they did not examine the effect on BP of weight loss among normal weight adolescents [29]. Further intervention research involving normal weight children would provide more insights into this discussion. While mechanisms underlying these differences are still being researched, recent evidence also indicates that the adipose tissue-derived hormone, leptin, may be a potentially important mediator in linking the effect of adiposity on BP [28]. Our results do suggest that small reductions in weight among obese children may lead to reductions in their BP.

The present study observed an inverse association between physical activity and SBP and DBP in adolescent boys. A number of observational and intervention studies have investigated associations between PA and BP in children and adolescents. Kelley et al. combined the results of 12 randomized trials and concluded that PA leads to a small but statistically significant reduction in BP [30]. Using accelerometer-measured PA data from the NHANES, Mark et al. described a modest dose–response relation of total PA and moderate-to-vigorous intensity PA with hypertension [31]. Leary et al. found a similar inverse association between total PA and SBP; however, when they compared the volume (duration and frequency) versus the intensity (vigorousness) of PA, they found that moderate to vigorous PA did not further reduce BP beyond the reduction achieved by a higher volume of PA, suggesting the volume of PA may be more important than the intensity of PA in reducing BP [15,16]. Since PA levels defined in the CHMS did not distinguish between the volume and intensity of PA, we were unable to assess their individual effects on BP. Nevertheless, our study identified inactive or moderately active adolescent boys as at risk of increased SBP and DBP, suggesting that PA should be intensified to mediate the negative effect on BP of other risk factors, and be promoted particularly for girls as evidence suggests that girls are less likely to participate in moderate to vigorous PA than boys [32]. Current clinical guidelines recommend that vigorous PA such as participation in competitive sports should only be limited in the presence of uncontrolled stage 2 hypertension, which is above 99^th^ percentile for gender, age and height [7].

Research shows that both genetic and environmental factors significantly influence BP and the development of hypertension during childhood [17]. Our results show that the strength of associations between BMI and BP was not modified by the presence of FHH. What our cross-sectional study was unable to address, however, is whether children with FHH are more susceptible to elevated BP due to increased BMI or decreased PA; more research is needed to explore effects of other risk factors in the presence of FHH so that targeted intervention can be designed for this genetically predisposed population. Interestingly, in future studies, increases in environmentally induced high blood pressure may serve to mask the association of blood pressure risk and genetic factors, for which FHH is a proxy.

Finally, the inverse relationship of post-secondary parental education with SBP in children, especially girls, is similar to previous findings in which BP in girls was related to socioeconomic variables in addition to anthropometric factors [33]. However, living in a low income family situation did not appear to increase risk of high BP, and was even associated with lower BP in children in this study. While some recent epidemiological studies from western developed countries have pointed that SES is inversely associated with childhood obesity, evidence on the relationship of SES with BP in childhood is limited [18]. Howe et al. in a recent study indicated important socioeconomic inequalities in obesity and cardiovascular risk factors including childhood BP in children; however, the SES indicator used in their study was maternal education [34]. In Canada, findings on SES and its relation with childhood obesity are conflicting [35,36]. In fact, we have noticed in our data that prevalence of obesity is lower in the lowest income (9.6 %) than middle to high income groups (13.9 %). With ongoing data collection for the next cycle of the CHMS, more studies with larger survey samples, detailed information on SES and inclusion of more variables (i.e. dietary, psychological factors) are expected to verify our findings.

The present study has several strengths. First, the CHMS is a high quality survey with standardized BP measurement for a nationally representative sample. Second, the sample of children aged 6 to 17 years was large enough to achieve sufficient statistical power to assess the independent effects of multiple variables. Limitations of our study include the cross-sectional survey design, which does not allow us to make casual inferences about BP and its determinants. However, reverse causation is unlikely. Another limitation is related to oscillometric measured BP, which may have underestimated DBP when compared with the US reference data using auscultatory method. However, the accuracy of the device has been validated by the company for its use in adults and children, and checked regularly by comparing with a T tube connected mercury sphygmomanometer for comparable readings for SBP and DBP [37,38]. Finally, dietary factors have been important in the prevention and treatment of hypertension in adults, especially intakes of sodium and potassium [39,40]. However, measures estimating these nutrient intakes are no available in cycle 1 of the CHMS.

## Conclusions

Due to the epidemic of childhood obesity in Canada and worldwide, increasing attention is being paid to the cardiovascular health of children and adolescents. Our study demonstrates the early impact of excess adiposity, insufficient PA, FHH and SES on BP and suggests that early interventions to reduce childhood obesity can, among other things, reduce exposure to prolonged blood pressure elevation and the future risk of cardiovascular disease. Guardians and/or physicians should pay close attention to weight gain, especially severe weight gain, in both children and adolescents. Public health planners and health care decision makers should consider the best means to ensure that children of all ages benefit from sufficient PA.

## Abbreviations

BP: blood pressure; PA: physical activity; FHH: family history of hypertension; SES: socioeconomic status; SBP: systolic BP; DBP: diastolic BP; BMI: body mass index.

## Competing interests

The authors declare that they have no competing interests.

## Author’s contributions

All authors were involved in designing the research, YS analysed the data, MDG advised on the analysis, MDG and HM gave advice and comments on interpretation, all authors were involved in writing the manuscript and all authors have read and approved the final manuscript.

## Pre-publication history

The pre-publication history for this paper can be accessed here:

http://www.biomedcentral.com/1471-2458/12/388/prepub
